# Bilibili, TikTok, and YouTube as sources of information on gastric cancer: assessment and analysis of the content and quality

**DOI:** 10.1186/s12889-023-17323-x

**Published:** 2024-01-02

**Authors:** Menghui Wang, Nan Yao, Jianming Wang, Wenjuan Chen, Yaobin Ouyang, Chuan Xie

**Affiliations:** 1 Department of Gastroenterology, The First Affiliated Hospital of Nanchang University, 17 Yong Waizheng Street, Donghu District, Nanchang, 330006 Jiangxi Province China; 2 Huan Kui College of Nanchang University, Nanchang, Jiangxi Province 330006 China; 3 Queen Mary College of Nanchang University, Nanchang, Jiangxi Province 330006 China; 4 Public Health College of Nanchang University, Nanchang, Jiangxi Province 330006 China; 5 Department of Oncology, Mayo Clinic, Rochester, MN 55905 USA

**Keywords:** Gastric cancer, Quality, Online video, The public, Social media

## Abstract

**Background:**

Gastric cancer has attracted widespread attention on social media due to its high incidence and severity. The Bilibili, TikTok, and YouTube video-sharing platforms have received considerable interest among general health consumers. Nevertheless, it remains unclear whether the information in videos on these platforms is of satisfactory content and quality.

**Methods:**

A total of 300 eligible videos related to gastric cancer were screened from three video-sharing platforms, Bilibili, TikTok, and YouTube, for assessment and analysis. First, the basic information presented in the videos was recorded. Next, we identified the source and content type of each video. Then, the Global Quality Scale (GQS), Journal of the American Medical Association (JAMA), and Modified DISCERN were used to assess the educational content and quality of each video. A comparative analysis was undertaken of the videos procured from these three sources.

**Results:**

We identified six categories of uploaders of the 300 videos: 159 videos (53%) were uploaded by health professionals, 21 videos (7%) by users in science communications, 29 videos (9.67%) by general users, 27 videos (9%) from news agencies, 63 videos (12%) by nonprofit organizations, and one video (0.33%) by a for-profit organization. In terms of the content types of the 300 videos, we identified five distinct categories. There were 48 videos (16%) on early signals, 12 videos (4%) on late symptoms, 40 videos (13.33%) on etiologies and causations, 160 videos (53.33%) on scientific introductions, and 40 videos (13.33%) on treatment methods. The overall quality of the videos was evaluated by the GQS, JAMA, and Modified DISCERN and was found to be medium, with scores of 2.6/5, 2.41/4, and 2.71/5 points, respectively.

**Conclusions:**

This innovative study demonstrates that videos on social media platforms can help the public learn about early signals, late symptoms, treatment methods, etiologies and causations, and scientific introductions of gastric cancer. However, both the content and quality of uploaded recordings are inadequate currently. More efforts should be made to enhance the content and quality of videos on gastric cancer and to increase public awareness.

**Supplementary Information:**

The online version contains supplementary material available at 10.1186/s12889-023-17323-x.

## Introduction

Gastric cancer is a significant global health issue, with millions of new cases diagnosed worldwide each year [1, 2]. According to the International Agency for Research on Cancer (IARC) GLOBOCAN project, gastric cancer accounted for more than 7.69 million newly diagnosed cases in 2020 [3]. While there has been a downwards trend in the incidence and mortality of gastric cancer for the past five decades [4], the disease still results in a severe global burden for its prevention and treatment [2, 5]. Consequently, the diagnosis and treatment of gastric cancer have become a critical priority for both medical professionals and patients alike.

Social media platforms are innovative, interactive, social networks where media practitioners can share information, express opinions, and exchange experiences in various ways, while the public can access, learn, and exchange knowledge [6, 7]. In recent years, video content dissemination has replaced traditional text-based information as the mainstream approach to information dissemination on social media. Especially during the recent COVID-19 pandemic, social media has had a greater impact on patients than ever before [8]. Social media platforms, including Bilibili, TikTok, and YouTube, play a crucial role in disseminating information about gastric cancer and providing public access to medical knowledge related to this disease. Bilibili, TikTok, and YouTube are all popular video-sharing platforms with a global user base [9]. The primary source of video content on these platforms is user-generated original videos or republished videos. As a result, these platforms exhibit a wealth of diverse content, and users can engage with videos through various means, such as comments, likes, and other interactive features [10]. Furthermore, these platforms possess both social and commercial dimensions [11]. The comparison of differences among the three online video platforms Bilibili, TikTok, and YouTube is described in detail in Table S1. These characteristics establish Bilibili, TikTok, and YouTube as popular and diverse video-sharing platforms. However, due to limitations associated with the public's level of education and the accuracy of these social media platforms, disseminating information related to gastric cancer through these social media channels can lead to uneven or misleading content [12, 13]. Hence, to provide the public with a more comprehensive and reliable platform for learning about gastric cancer-related information, we used the Global Quality Scale (GQS), Journal of the American Medical Association (JAMA), and Modified DISCREN system rating for online gastric cancer videos currently available on social media [14–16].

Currently, no study has been identified that analyses the status quo f gastric cancer video online, and the public’s knowledge of gastric cancer. Given the increasing public interest in gastric cancer, it is crucial to evaluate the effectiveness of media platforms in disseminating information about the disease. Therefore, our study analysed and rated videos on gastric cancer knowledge available on popular media platforms. Ultimately, this study aims to enhance the development of social media to provide more comprehensive and suitable directions for the public to access and learn about gastric cancer-related information.

## Materials and methods

### Search strategy

Videos were sought from three platforms, namely, Bilibili (www.bilibili.com), TikTok (Chinese version: www.douyin.com), and YouTube (www.youtube.com) to determine the impact of gastric cancer related information on the public on three video platform. The time limit for screening videos was up to March 31st, 2023. Video retrieval was performed on a single day, April 1st, 2023, to reduce the bias incurred by newly uploaded videos. Then, the start date of our analysis is April 2nd, 2023. The search term was "gastric cancer." The search history of the three platforms was deleted immediately before searching. Based on each video-sharing platform's global rankings determined by algorithmic computations, we watched videos one by one from the most highly ranked to the lowest. Detailed information concerning all the analysed videos was recorded for further analysis.

Three investigators (Chen WJ, Yao N, and Ouyang YB) independently reviewed and assessed the aforementioned videos. If any disparities or disagreements arose among the three investigators, two authors (Wang JM and Wang MH) were available to deliberate and establish an agreed-upon conclusion.

### Video selection criteria

The videos analysed in this study were taken from Bilibili, TikTok, and YouTube and were listed in the default combined order of the platforms. The inclusion criteria for the videos were 1) Chinese and English languages 2) all types of videos related to gastric cancer but content about scientific introductions, treatment methods, early signals, late symptoms, etiologies and causations of gastric cancer. Duplicate videos, advertisements, and irrelevant content were excluded. Finally, the first 100 videos on each platform that met the inclusion criteria were selected for further analysis [17, 18].

### Collection of video features

A total of 300 videos were analysed for this study. In detail, TikTok, Bilibili and YouTube each included 100 videos each for analysis. The collected data included the video title, source (health professionals, general users, science communications, news agencies, nonprofit organizations, and for-profit organizations) (Table S5), upload platform, upload time, duration, views, likes, comments, and content (early signals, late symptoms, etiologies and causations, scientific introductions, and treatment methods) as well as the use of the GQS, JAMA, and Modified DISCERN for video assessment.

The sources of the videos were categorized into two main groups: individual and organizational users. Individual users included health professionals such as doctors and nurses, general users, and users in science communications, such as popular science writers [19]. Organizational users included news agencies, nonprofit organizations, and for-profit organizations. Nonprofit organizations were defined as those with a focus on collective, public, or social benefit and included public hospitals [20], while for-profit organizations were those that pursued commercial interests [21]. Content analysis was performed across five key aspects related to gastric cancer: early signals, late symptoms, etiologies and causations, scientific introductions, and treatment methods.

### Assessment of each video

**Fig. 1:**
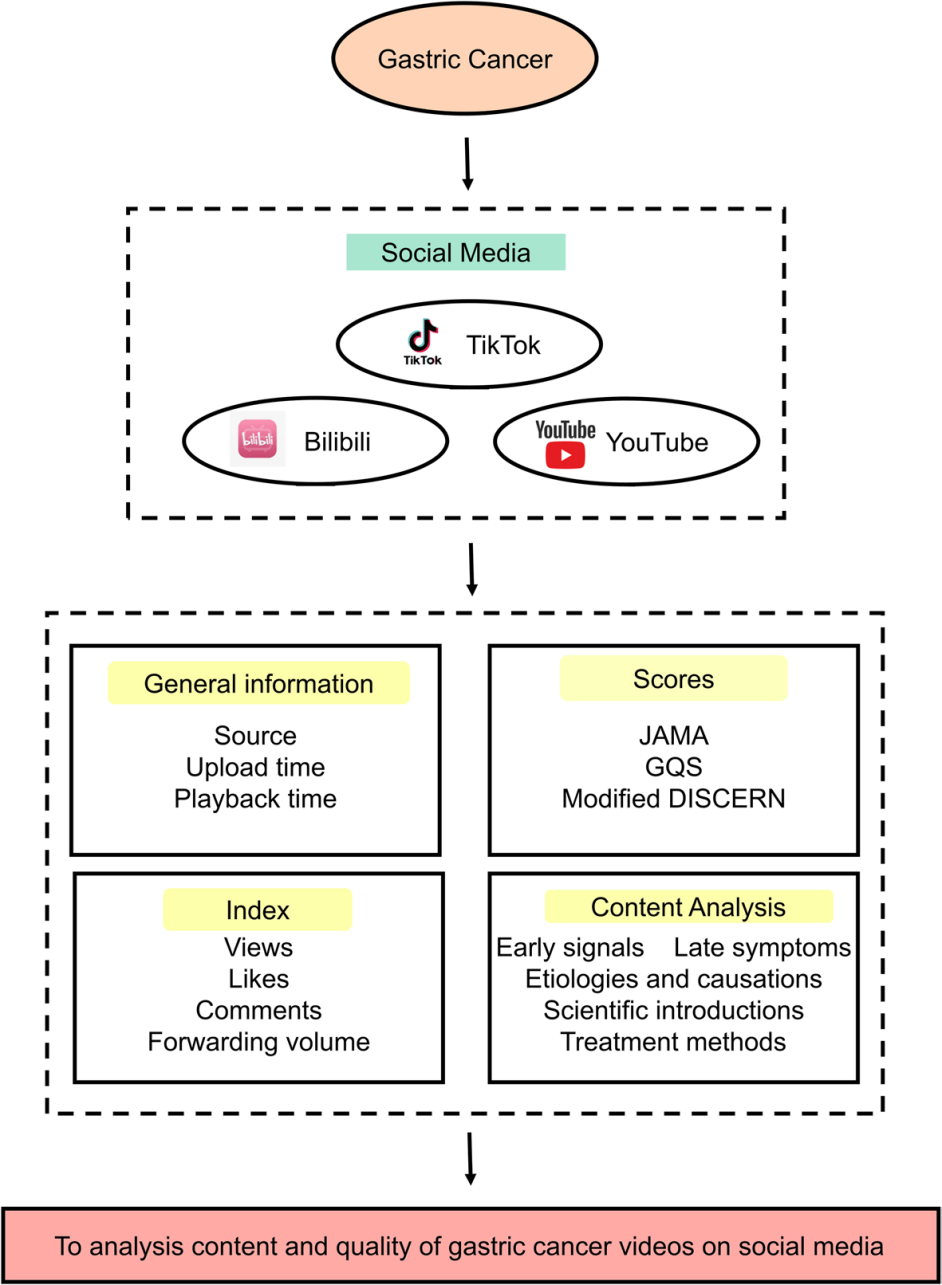
The framework of the study

In this study, a content assessment of the videos was conducted utilizing three commonly employed standard scales: the GQS (Table S2), JAMA (Table S3), and Modified DISCERN (Table S4). These scales were employed to evaluate the quality and effectiveness of the analysed videos (Fig. 1).

The present study employed a multifaceted approach to assess the educational content quality and reliability of the videos investigated. Specifically, the GQS was employed to evaluate dimensions such as quality, flow, comprehensiveness and usefulness for patients, with scores ranging from 1 (indicating poor quality) to 5 (indicating excellent flow and quality) [22]. For the assessment of video reliability, JAMA benchmarks were utilized, which encompass four key criteria: authorship, attribution, currency, and disclosure. In other words, the video content was evaluated based on the extent to which it 1) provided authorship information; 2) listed copyright information and references/sources; 3) included the initial date and subsequent updates; and 4) disclosed any potential conflicts of interest, funding, sponsorship, advertising support or video ownership. Each criterion was awarded 1 point, resulting in a total maximum score of 4 points [23]. To further assess the quality and reliability of the videos, a modified version of the DISCERN tool was used. This tool comprised five questions, with a score of 1 point given for each affirmative answer and a score of 0 points given for each negative response [24]. Specifically, the five questions focused on whether 1) the video was clear, concise and understandable; 2) the information sources were reliable; 3) the information presented was balanced and unbiased; 4) additional sources of information were provided for patient reference; and 5) areas of uncertainty or controversy were appropriately addressed.

### Statistical analysis

For the data analysis, we utilized the Statistical Package for the Social Sciences 22 (IBM, Armonk, NY, USA). To assess the normality of the data, the Shapiro‒Wilk test was employed. If the quantitative data conformed with a normal distribution, then the data were presented as the mean ± standard deviation (‾x ± SD). Descriptive statistics, including mean, standard deviation, frequency, minimum, and maximum values, were calculated. Descriptive statistics are presented as the mean (minimum, maximum). The Kruskal‒Wallis test, a nonparametric statistical test, was performed to evaluate whether there were significant differences among three or more groups of independent variables. Subsequently, the Dunn-Bonferroni methodology was utilized for pairwise comparison in the case of significant Kruskal‒Wallis results. Spearman's test was executed to examine the correlation between independent variables. The kappa coefficient was applied to evaluate interrater agreement. Finally, the results were assessed through a 95% confidence interval with a significance level of *P* < 0.05. The Bonferroni adjustment was automatically conducted in SPSS (version: IBM SPSS Statistics22) by multiplying Dunn's P value by the number of comparisons.

## Results

### Overview of the video screening process

**Fig. 2:**
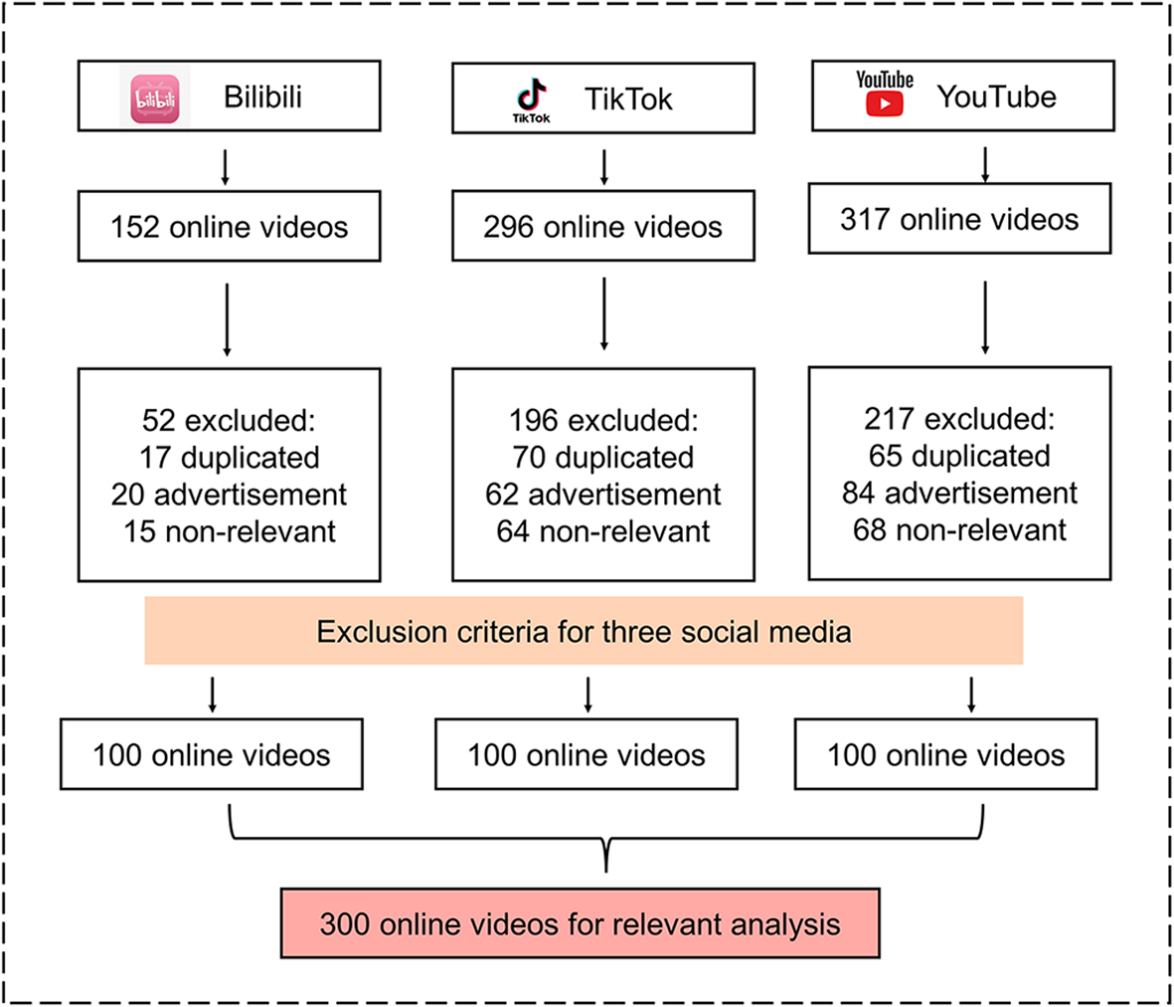
Flowchart of filtering gastric cancer for further analysis

A total of 765 gastric cancer-related videos were retrieved from the three platforms Bilibili, TikTok, and YouTube. After removing 152 duplicates, 166 advertisements, and 147 irrelevant videos, 300 eligible videos were included for further analysis (Fig. 2).

### General information and index of online videos

**Fig. 3:**
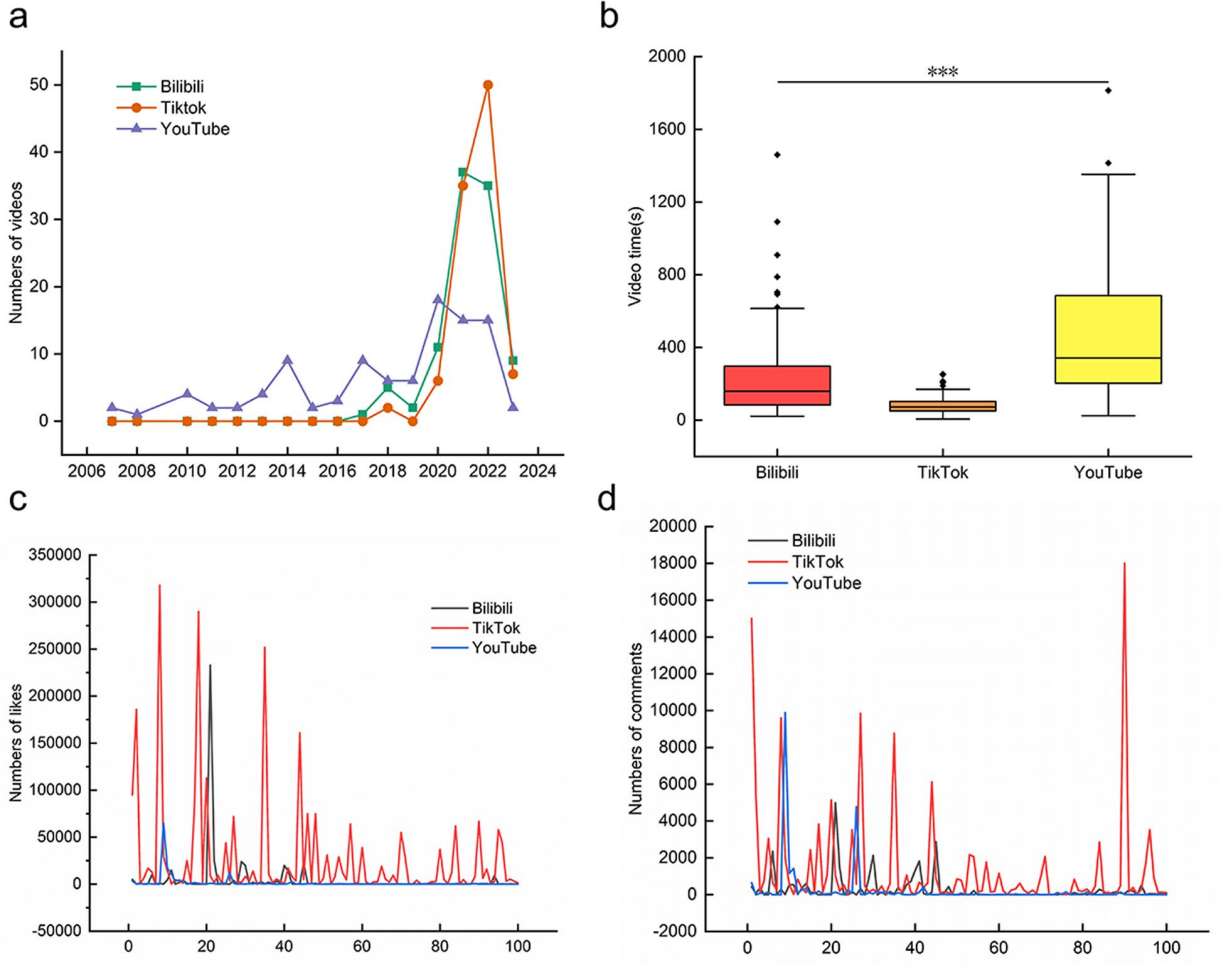
General information on gastric cancer‑related videos sourced from the three video‑sharing platforms. **a** A line chart shows 300 eligible gastric cancer-related videos released between 2007 and 2023 that met the inclusion criteria. **b** The playback time of gastric cancer-related videos on the three video‑sharing platforms. **c** The line chart shows the number of likes for the three platforms. **d** The line chart shows the number of comments for the three platforms

The online videos selected were uploaded between May 15, 2007, and March 30, 2023 (Fig. 3a). From 2017 to 2023, videos uploaded on the social networking platform Bilibili included 1 video (1%) uploaded in 2017, 5 videos (5%) in 2018, 2 videos (2%) in 2019, 11 videos (11%) in 2020, 37 videos (37%) in 2021, 35 videos (35%) in 2022, and 9 videos (9%) in 2023. For the social media platform TikTok, 2 videos (2%) were uploaded in 2018, 6 videos (6%) were uploaded in 2020, 35 videos (35%) were uploaded in 2021, 50 videos (50%) were uploaded in 2022, and 7 videos (7%) were uploaded in 2023. Videos uploaded to YouTube were viewed between 2007 and 2023. 2 videos (2%) were uploaded in 2007, 1 video (1%) in 2008, 4 videos (4%) in 2010, 2 videos (2%) in 2011, 2 videos (2%) in 2012, 4 videos (4%) in 2013, 9 videos (9%) in 2014, 2 videos (2%) in 2015, 3 videos (3%) in 2016, 9 videos (9%) in 2017, 6 videos (6%) in 2018, 6 videos (6%) in 2019, 18 videos (18%) in 2020, 15 videos (15%) in 2021, 15 videos (15%) in 2022, and 2 videos (2%) in 2023. The duration of 100 gastric cancer videos on Bilibili was 240.6 (21,1460) seconds. In comparison, the duration of 100 gastric cancer videos on TikTok was 84.1 (6, 253) seconds, and the duration of 100 gastric cancer videos on YouTube was 462.1 (24, 1814) seconds. A statistically significant difference (*P* < 0.001) was found between the duration of videos played on Bilibili and TikTok and the video watch time on YouTube (Fig. 3b), possibly due to the configuration of the platforms. Moreover, videos on TikTok garnered more likes (Fig. 3c) and comments (Fig. 3d) than those on Bilibili and YouTube.

The general information and index of online video in the three platforms is shown in Table 1.

**Table 1:** Basic index and scores of videos about gastric cancer on three different platforms from different time periods

	Index	Bilibili		TikTok		YouTube	
		Mean ± SD	(MIN, MAX)	Mean ± SD	(MIN, MAX)	Mean ± SD	(MIN, MAX)
2007–2010	Views	/	/	/	/	80,095 ± 72,598	(43,796, 116,394)
	Number of likes	/	/	/	/	113.5 ± 209	(9, 218)
	Number of comments	/	/	/	/	38 ± 50	(13, 63)
	Number of forwards	/	/	/	/	/	/
	Duration (s)	/	/	/	/	213.5 ± 209	(24, 233)
	Time since upload (d)	/	/	/	/	5119.5 ± 575	(4832, 5407)
2011–2014	Views	/	/	/	/	55,876.46 ± 124,511	(8290, 1197)
	Number of likes	/	/	/	/	315.38 ± 1880	(0, 1880)
	Number of comments	/	/	/	/	45.77 ± 254	(0, 254)
	Number of forwards	/	/	/	/	/	/
	Duration (s)	/	/	/	/	368 ± 1112	(85, 1197)
	Time since upload (d)	/	/	/	/	3219 ± 4162	(122, 4284)
2015–2018	Views	20.83 ± 35	(10, 45)	/	/	582,925.7 ± 4,797,784	(2653, 4,800,437)
	Number of likes	19.83 ± 90	(0, 90)	2576	(2576, 2576)	5836.4 ± 65,000	(0, 65,000)
	Number of comments	31.83 ± 90	(6, 95)	291	(291, 291)	819.93 ± 9891	(0, 9891)
	Number of forwards	7397.33 ± 29,378	(622, 30,000)	333	(333, 333)	/	/
	Duration (s)	107.67 ± 74	(78, 152)	59	(59, 59)	501.53 ± 1020	(103, 1123)
	Time since upload (d)	1833.17 ± 371	(1767, 2138)	1739	(1739, 1739)	2054.4 ± 1640	(1344, 2984)
2019–2023	Views	19,128.5 ± 206,984	(16, 207,000)	/	/	53,474.57 ± 427,878	(430,565, 2687)
	Number of likes	504.89 ± 9302	(0, 9302)	26,785.6 ± 317,877	(123, 318,000)	829.55 ± 12,000	(0, 12,000)
	Number of comments	70.41 ± 504	(0, 504)	1348.23 ± 17,994	(6, 18,000)	135.13 ± 4777	(0, 4777)
	Number of forwards	56.08 ± 1200	(0, 1200)	7903.83 ± 219,971	(29, 220,000)	/	/
	Duration (s)	788 ± 767	(21, 788)	84.14 ± 247	(6, 253)	484.86 ± 1784	(30, 1814)
	Time since upload (d)	541.08 ± 1056	(3, 1059)	467.85 ± 1842	(2, 1844)	1260.96 ± 5773	(27, 5800)

### Content analysis of online videos

**Fig. 4:**
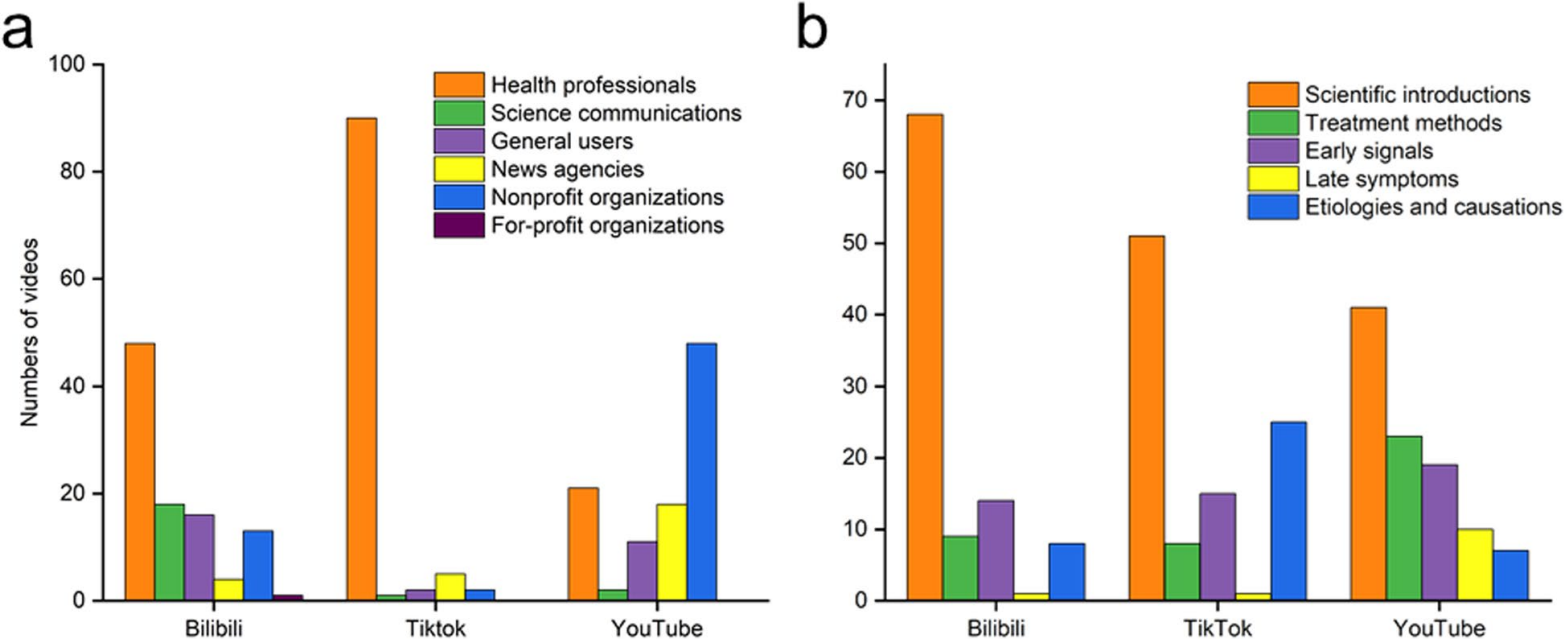
**a** The bar chart shows video sources for the three platforms. **b** The bar chart shows the content types of the video on the three platforms

The one hundred videos for each platform analysed in this study were contributed by a variety of sources, including health professionals, science communications, general users, news agencies, nonprofit organizations, and for-profit organizations. The data indicate that among the three platforms assessed, Bilibili boasted the highest number of videos released by health professionals, with 48 videos (48%) published. Health professionals contributed the highest number of TikTok videos, with a total of 90 videos (90%) released. Nonprofit organizations posted the highest number of YouTube videos, constituting 48% of the total (Fig. 4a). Upon reviewing 300 videos that met the screening criteria for gastric cancer, the videos were classified into five categories: early signals, late symptoms, etiologies and causations, scientific introductions, and treatment methods. The data revealed that scientific introductions (n = 160, 53.33%) videos made up the largest proportion, followed by early signals (n = 48, 16%) and, etiologies and causations and treatment methods (n = 40, 13.33%) holding identical proportions and late symptoms (n = 12, 4%) (Fig. 4b). Notably, the number of scientific introductions videos related to gastric cancer was the highest across all three platforms. Early signals held the second position on Bilibili, etiologies and causations held the second position on TikTok, and treatment methods held the second position on YouTube (Table 2).

**Table 2:** Descriptions of video source and content

	Source	Content	Videos (n)
Individual users	Health professionals	Scientific introductions	88
		Treatment methods	15
		Early signals	27
		Late symptoms	3
		Etiologies and causations	26
	Science communications	Scientific introductions	15
		Treatment methods	2
		Early signals	2
		Late symptoms	1
		Etiologies and causations	1
	General users	Scientific introductions	17
		Treatment methods	4
		Early signals	5
		Late symptoms	1
		Etiologies and causations	2
Organizational users	News agencies	Scientific introductions	13
		Treatment methods	1
		Early signals	5
		Late symptoms	3
		Etiologies and causations	5
	Nonprofit organizations	Scientific introductions	26
		Treatment methods	18
		Early signals	9
		Late symptoms	4
		Etiologies and causations	6
	For-profit organizations	Scientific introductions	1
		Treatment methods	0
		Early signals	0
		Late symptoms	0
		Etiologies and causations	0

Upon analysis of the source of uploaded videos, health professionals were found to have contributed the greatest number of science communications videos (n = 88, 55.35%), followed by nonprofit organizations (n = 26, 41.27%). Among the science communication videos, scientific introductions were the predominant topic (n = 15, 71.43%), followed by treatment methods (n = 2, 9.52%) and early signals (n = 2, 9.52%). General users posted videos in which scientific introductions were also the most common theme (n = 13, 48.15%), followed by early signals (n = 5, 17.86%). In contrast, news agencies predominantly uploaded scientific introductions videos (n = 17, 58.62%), with etiologies and causations and early signals both accounting for the second largest number of videos (n = 5, 17.24%). Nonprofit organizations also featured scientific introductions prominently (n = 26, 41.27%), while treatment methods (n = 18, 28.57%) occupied the second highest number of videos. Finally, scientific introductions were the sole topic presented in videos uploaded by for-profit organizations (n = 1, 100%).

### Quality analysis of online videos

**Fig. 5:**
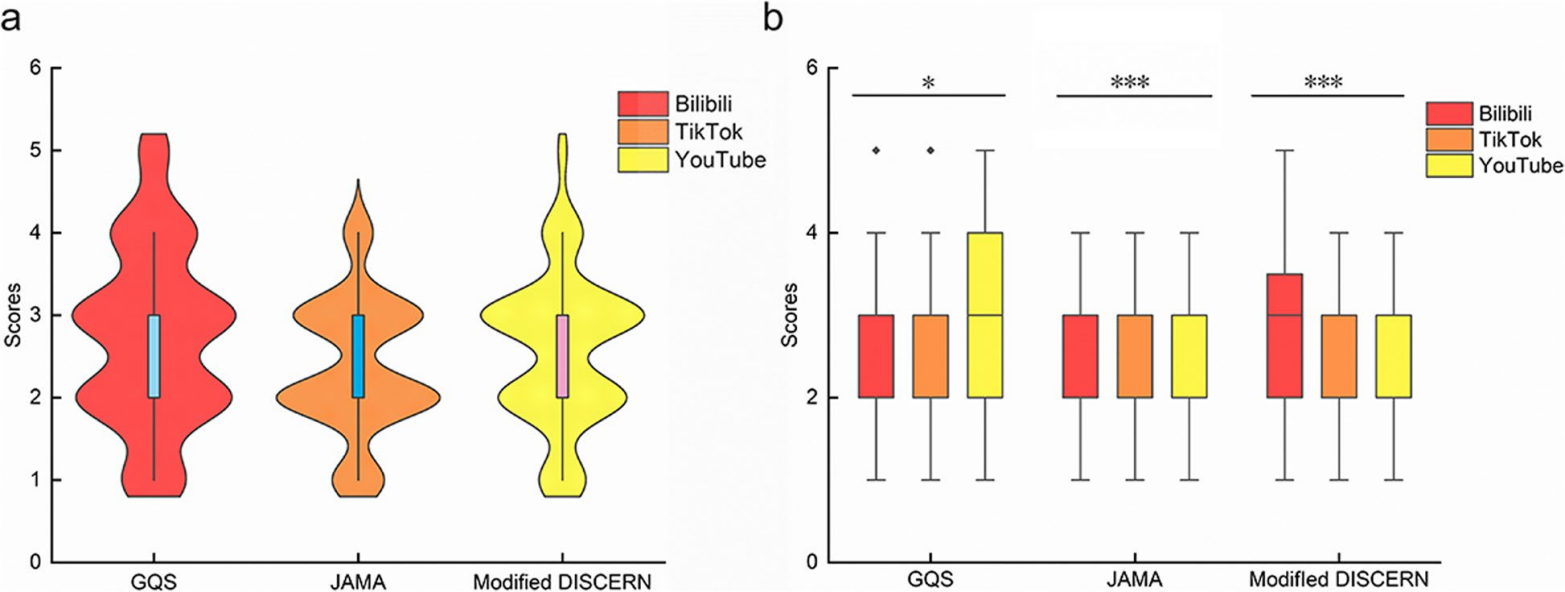
**a** The violin graph shows the scores of GQS, JAMA, and Modified DISCERN on three social platforms. **b** The box graph shows a comparison of GQS, JAMA, and Modified DISCERN scores on three social platforms

The included videos on the three distinct platforms underwent scoring procedures based on the respective scoring schemes. The cross-platform analysis resulted in an overall GQS of 2.6 (1,5) for the included videos. Furthermore, an overall Journal of American Medical Association (JAMA) score of 2.41 (1,4) was identified. The Modified DISCERN score for the compiled videos in the study was 2.71 (1,5) (Fig. 5a). Statistical analyses revealed that videos on Bilibili and YouTube tended to obtain higher GQS, JAMA, and Modified DISCERN scores compared to those shared on TikTok. The observed differences were statistically significant (*P* < 0.05, *P* < 0.001, *P* < 0.001, respectively) (Fig. 5b). The detailed scoring allocations across the three scoring criteria are summarized in Table 3.

**Table 3:** Comparison of source, content and scores of videos about gastric cancer on three different platforms

	Total	Bilibili	TikTok	YouTube	
**Number**	300	100	100	100	*P* value
**Source**	N (%)	N (%)	N (%)	N (%)	< 0.001
Health professionals	159 (53%)	48 (48%)	90 (90%)	21 (21%)	
Science communications	21 (7%)	18 (18%)	1 (1%)	2 (2%)	
General users	29 (9.67%)	16 (16%)	2 (2%)	11 (11%)	
News agencies	27 (9%)	4 (4%)	5 (5%)	18 (18%)	
Nonprofit organizations	63 (12%)	13 (13%)	2 (2%)	48 (48%)	
For-profit organizations	1 (0.33%)	1 (1%)	0	0	
**Content**	N (%)	N (%)	N (%)	N (%)	< 0.001
Early signals	48 (16%)	14 (14%)	15 (15%)	19 (19%)	
Late symptoms	12 (4%)	1 (1%)	1 (1%)	10 (10%)	
Etiologies and causations	40 (13.33%)	8 (8%)	25 (25%)	7 (7%)	
Scientific introductions	160 (53.33%)	68 (68%)	51 (51%)	41 (41%)	
Treatment methods	40 (13.33%)	9 (9%)	8 (8%)	23 (23%)	
**GQS**	2.6 (1,5)	2.76 (1,5)	2.14 (1,5)	2.9 (1,5)	0.046
1 score	36 (12%)	14 (14%)	17 (17%)	5 (5%)	
2 score	95 (31.67%)	29 (29%)	37 (37%)	29 (29%)	
3 score	101 (33.67%)	34 (34%)	28 (28%)	39 (39%)	
4 score	50 (16.67%)	13 (13%)	12 (12%)	25 (25%)	
5 score	18 (6%)	10 (10%)	6 (6%)	2 (2%)	
**JAMA**	2.41 (1,4)	2.47 (1,4)	2.22 (1,4)	2.54 (1,4)	< 0.001
1 score	39 (13%)	14 (14%)	20 (20%)	5 (5%)	
2 score	130 (43.33%)	39 (39%)	50 (50%)	41 (41%)	
3 score	108 (36%)	33 (33%)	26 (26%)	49 (49%)	
4 score	23 (7.67%)	14 (14%)	4 (4%)	5 (5%)	
**Modified DISCERN**	2.71 (1,5)	2.79 (1,5)	2.53 (1,5)	2.82 (1,5)	< 0.001
1 score	34 (11.33%)	14 (14%)	19 (19%)	1 (1%)	
2 score	96 (32%)	24 (24%)	46 (46%)	26 (26%)	
3 score	130 (43.33%)	37 (37%)	29 (29%)	64 (64%)	
4 score	33 (11%)	19 (19%)	6 (6%)	8 (8%)	
5 score	7 (2.33%)	6 (6%)	0	1 (1%)	

## Discussion

### Principal findings

Gastric cancer is a prevalent form of cancer caused by various factors, including Helicobacter pylori infection [25]. The high costs and limited survival duration associated with treating gastric cancer significantly impact patients’ lives [26], causing widespread concern within society regarding health and well-being. A systematic review and meta-analysis showed significant geographical variation in the incidence and mortality rates of gastric cancer [27]. Previous studies have shown that the incidence and mortality of gastric cancer vary in different regions, such as East Asia and Western countries [28–30]. The scientific introductions videos from the three platforms also mentioned that different regions have different incidences and mortality rates for gastric cancer. In this study, Bilibili and TikTok were found to have higher-quality gastric cancer videos than YouTube. This can be attributed to the higher level of public attention devoted to the disease in the local community. Given the complexity of the causation and treatment modalities involved in gastric cancer, disseminating knowledge about the disease through social media is crucial.

The role and significance of social media platforms in modern society cannot be ignored. These platforms enable people to quickly access and share comprehensive information [31, 32]. However, social media also poses potential risks to human health and has several negative consequences [33]. The pivotal role and significance of social media in facilitating information dissemination are well established, providing unparalleled expediency in access to the latest, most comprehensive and readily available information [34]. In times of disasters or emergency events, social media platforms can significantly improve the speed and efficiency of information conveyance, enabling people to promptly understand the current situation and respond effectively [35]. Past studies have revealed that social media has an increasingly critical role in infectious disease modelling, thereby enhancing the accuracy of disease forecasting models [36]. In addition, social media provides individuals with opportunities to express their opinions and to present diverse and democratic information to the public. By engaging with social media communication, the public's attention to health can be increased, resulting in better health literacy and the elimination of misinformation [37]. However, social media also faces challenges concerning information credibility and authenticity. People often find it challenging to differentiate between authentic and misleading information. Even some for-profit organizations use some functions of social media to exaggerate facts, leading to false information that causes misinformation among the public [38].

An analysis of three distinct social media platforms revealed significant differences in attitudes towards gastric cancer between China and the rest of the world. Specifically, the social media platform Bilibili featured 68 science-related videos and 32 professional videos about gastric cancer, while TikTok showcased 51 popular science videos and 49 professional videos on the topic. In China, the most widely viewed popular science videos employed intriguing methods to educate the public on gastric cancer. For instance, popular science bloggers visually demonstrated the condition within the stomach via gastroscopy and endoscopic submucosal dissection (ESD), while others presented graphical analyses of real gastric cancer cases to educate the public on various treatment approaches. Notably, a unique approach incorporated 3D demonstration technology to convey the progression of gastric cancer to the public. Moreover, these popular science videos informed the public that gastric cancer can be effectively prevented by abstaining from tobacco and alcohol use, getting proper rest, and undergoing regular check-ups. The videos recommended consuming certain foods, such as chili peppers and garlic, to reduce the risk of gastric cancer while noting that high salt intake increases the chances of developing the disease. Importantly, these videos serve to heighten public awareness of gastric cancer prevention and improve treatment efficacy. By understanding the fundamentals of the condition, patients' anxiety is often minimized, enhancing the therapeutic impact of gastric cancer treatment. Timely interventions can be initiated after the symptoms and warning signs are identified, facilitating the possibility for early detection, accurate diagnosis, and appropriate treatment. Finally, these popular science videos underline the significance of cancer prevention measures through lifestyle adjustments and specific actions, such as the eradication of Helicobacter pylori. Many of these videos feature professional content published by health experts, enhancing their credibility. In China, a significant proportion of patients may not present with early warning symptoms of gastric cancer and may only experience abdominal pain, abnormal stools, nausea, vomiting, and other symptoms at later stages, with a severely diminished quality of life. While there are numerous treatment approaches to gastric cancer, the optimal method is dependent on the individual's situation, such as surgical intervention, radiotherapy, and targeted therapy. Causes of gastric cancer are influenced by factors such as diet, emotional state, environment, and genetics, among other variables.

The social media platform YouTube featured 41 science-related videos and 59 professional videos about gastric cancer. The symptoms and treatment of gastric cancer were the focus of popular science videos on YouTube. However, popular science videos mainly featured professional lectures with little creativity rather than engaging popular science techniques. In contrast to China, the United States has made substantial advancements in the prevention and treatment of gastric cancer through the incorporation of effective screening strategies such as Helicobacter pylori testing and endoscopy, which have reduced mortality rates [39]. Despite this, the report highlights a higher awareness of gastric cancer prevention among the Chinese population when compared to that of Americans, emphasizing the ongoing need to promote user education and knowledge dissemination to bolster overall scientific literacy and awareness of gastric cancer globally.

According to the findings of the present research, the social media platforms Bilibili and TikTok and the global-sharing platform YouTube spread essential information about gastric cancer. The research identified a plethora of crucial information related to gastric cancer, including early signals symptoms, treatment methods, etiologies and causations, and applications of scientific introductions. The remarkable levels of engagement based on metrics such as comments and likes on these social media videos highlight the potential for these platforms to effectively disseminate vital gastric cancer-related knowledge among the public, resulting in increased awareness and emphasis on healthy dietary habits [40, 41]. It is noteworthy that videos on the Bilibili and TikTok platforms predominantly featured content created by health care professionals, indicating that medical practitioners in China attach great significance to gastric cancer prevention and treatment awareness. In contrast, nonprofit organizations were responsible for the majority of video publishing on the YouTube platform.

The quality of the videos analysed in this study was assessed using the GQS, JAMA, and Modified DISCERN rating scales, all of which yielded moderate scores (2.6/5, 2.4/4, and 2.7/5, respectively). These findings suggest that the overall quality of online gastric cancer-related videos is suboptimal. This gap between high- and low-quality information is due to both objective and subjective factors in health care, making it challenging for the public to distinguish between accurate and inaccurate information in today's intricate social media landscape [42]. To address this concern, efforts must be made to enhance the content and quality of gastric cancer videos and disseminate reliable information about the diagnosis, treatment, and underlying causes of the disease to the public in a more effective way. First, key users on the platform need to work to improve and ensure the content and quality of the videos [43, 44]. For example, key users may demand that the production process and content of the videos is as rigorous and accurate as possible. Second, regulatory agencies must closely monitor health-related video content and prevent the dissemination of misleading videos on the internet [45]. Finally, relevant governments, professional organizations, and experts should proactively counter misinformation or disseminate quality health-related information on social media. In addition, public consumers must selectively consume credible videos to improve their judgement, seek guidance from reliable sources, and consult with professionals to prevent or treat gastric cancer [46].

### Limitations

It is important to acknowledge the limitations of this study. The selection of videos was restricted to only the English and Chinese languages, overlooking the potential presence of informative gastric cancer videos in other languages, such as Korean and Japanese. Furthermore, the nature of video uploaders' control over the removal of uploads could potentially bias the research findings regarding audience search outcomes.

## Conclusions

This study provides reliable and valuable information for the public to understand the present state of gastric cancer-related online videos on social media platforms. The findings are conducive to enhancing the quality and content of these online videos and improving the public's accurate perception of gastric cancer. The outcomes unambiguously demonstrate the vital role played by social media platforms in disseminating essential knowledge pertaining to gastric cancer, ranging from early signals and late symptoms to etiologies and causations as well as scientific and technological advancements and treatment methods. Nonetheless, the scores achieved by the GQS, JAMA, and Modified DISCERN fall short of satisfactory levels. Therefore, it is imperative for professionals to acknowledge and address these issues in future releases of high-quality online videos. Furthermore, social media platform auditors should ensure accurate assessments to disseminate accurate knowledge about gastric cancer to the public.

### Supplementary Information

**Additional file 1: Table S1.** The comparison of Bilibili, TikTok, YouTube.

**Additional file 2: Table S2.** Global Quality Score (GQS) benchmark criteria.

**Additional file 3: Table S3.** The Journal of the American Medical Association (JAMA) benchmark criteria.

**Additional file 4: Table S4.** Modified DISCERN benchmark criteria.

**Additional file 5: Table S5.** Descriptions of video sources.

## Data Availability

The data sets generated during and/or analysed during this study are available from the corresponding author on reasonable request.
