# Supplementary material for: Bilibili, TikTok, and YouTube as sources of information on gastric cancer: assessment and analysis of the content and quality
Source: BMC Public Health. 2024 Jan 2;24:57. doi: 10.1186/s12889-023-17323-x (PMC10763378; doi:10.1186/s12889-023-17323-x)
Supplement: Supplementary file 1 — Additional file 1: Table S1. The comparison of Bilibili, TikTok, YouTube. [file 12889_2023_17323_MOESM1_ESM.docx]

Table S1. The comparison of Bilibili, TikTok, YouTube.

| Platforms | Position | functions |
| --- | --- | --- |
| Bilibili | Bilibili is a video platform in China that integrates entertainment and education. It is one of the ways for patients and doctors to understand and learn health-related knowledge. | Video, live, comment, like, forward, bullet screen. |
| TikTok | TikTok, a popular video-sharing social media platform, enables active learning through its short, condensed, and fun nature. | Video, live, comment, like, forward |
| YouTube | YouTube is the second largest social media platform and is increasingly being used by patients as a source of medical information due to its ease of access. | YouTube shorts, YouTube videos and live. |
