# Supplementary material for: Bilibili, TikTok, and YouTube as sources of information on gastric cancer: assessment and analysis of the content and quality
Source: BMC Public Health. 2024 Jan 2;24:57. doi: 10.1186/s12889-023-17323-x (PMC10763378; doi:10.1186/s12889-023-17323-x)
Supplement: Supplementary file 2 — Additional file 2: Table S2. Global Quality Score (GQS) benchmark criteria. [file 12889_2023_17323_MOESM2_ESM.docx]

Table S2. Global Quality Score (GQS) benchmark criteria.

| Score | Global Score Description |
| --- | --- |
| 1 score | poor quality, poor traffic, most information missing, not of any use to the patient |
| 2 score | Generally poor quality with poor flow, some information listed but many important topics lacked, of very limited use to patients |
| 3 score | medium quality with suboptimal flow, some of the main information was fully discussed but other information discussed insufficiently, somewhat helpful to patients. |
| 4 score | Good quality and generally goo flow, most of the relevant information is listed, but some topics not covered, useful to patients. |
| 5 score | Excellent quality and excellent flow, very useful for patients |
