# Supplementary material for: Bilibili, TikTok, and YouTube as sources of information on gastric cancer: assessment and analysis of the content and quality
Source: BMC Public Health. 2024 Jan 2;24:57. doi: 10.1186/s12889-023-17323-x (PMC10763378; doi:10.1186/s12889-023-17323-x)
Supplement: Supplementary file 3 — Additional file 3: Table S3. The Journal of the American Medical Association (JAMA) benchmark criteria. [file 12889_2023_17323_MOESM3_ESM.docx]

Table S3. The Journal of the American Medical Association (JAMA) benchmark criteria.

| Score* | Score component | |
| --- | --- | --- |
| 1 score | Authorship | Provided authorship information |
| 1 score | Attribution | Listed copyright information and references/sources |
| 1 score | Currency | Included the initial date and subsequent updates |
| 1 score | Disclosure | Disclosed any potential conflicts of interest, funding, sponsorship, advertising support or video ownership |

*The criteria of each aspect were scored separately, and 1 point was accumulated when the criteria were reached. A total reliability score ranging from 0 to 5 was obtained.
