# Supplementary material for: Bilibili, TikTok, and YouTube as sources of information on gastric cancer: assessment and analysis of the content and quality
Source: BMC Public Health. 2024 Jan 2;24:57. doi: 10.1186/s12889-023-17323-x (PMC10763378; doi:10.1186/s12889-023-17323-x)
Supplement: Supplementary file 4 — Additional file 4: Table S4. Modified DISCERN benchmark criteria. [file 12889_2023_17323_MOESM4_ESM.docx]

Table S4. Modified DISCERN benchmark criteria.

| Score* | Reliability Score |
| --- | --- |
| 1 score | The video was clear, concise and understandable |
| 1 score | The information sources were reliable |
| 1 score | The information presented was balanced and unbiased |
| 1 score | Additional sources of information were provided for patient reference |
| 1 score | Areas of uncertainty or controversy were appropriately addressed. |

*The criteria of each aspect were scored separately, and 1 point was accumulated when the criteria were reached. A total reliability score ranging from 0 to 5 was obtained.
