# Supplementary material for: Bilibili, TikTok, and YouTube as sources of information on gastric cancer: assessment and analysis of the content and quality
Source: BMC Public Health. 2024 Jan 2;24:57. doi: 10.1186/s12889-023-17323-x (PMC10763378; doi:10.1186/s12889-023-17323-x)
Supplement: Supplementary file 5 — Additional file 5: Table S5. Descriptions of video sources. [file 12889_2023_17323_MOESM5_ESM.docx]

Table S5. Descriptions of video sources.

| Source type | Source description |
| --- | --- |
| **Individual users** |  |
| Health professionals | Individuals who identify themselves as health professionals (eg, doctors and nurses) |
| General users | General users (eg, general health consumer) |
| Science communications | Individuals who are engaged in scientific communication (eg, popular science writers) |
| **Organizational users** |  |
| News agencies | News agencies and the press |
| Nonprofit organizations | Organizations operated for collective, public, or social benefit and public hospitals |
| Nonprofit organizations | Organizations that pursue commercial interests |
